# Use of Automated Thematic Annotations for Small Data Sets in a Psychotherapeutic Context: Systematic Review of Machine Learning Algorithms

**DOI:** 10.2196/22651

**Published:** 2021-10-22

**Authors:** Alexandre Hudon, Mélissa Beaudoin, Kingsada Phraxayavong, Laura Dellazizzo, Stéphane Potvin, Alexandre Dumais

**Affiliations:** 1 Centre de recherche de l'Institut Universitaire en Santé Mentale de Montréal Montréal, QC Canada; 2 Department of Psychiatry and Addictology Faculty of Medicine Université de Montréal Montréal, QC Canada; 3 Services et Recherches Psychiatriques AD Montréal, QC Canada; 4 Institut national de psychiatrie légale Philippe-Pinel Montréal, QC Canada

**Keywords:** psychotherapy, artificial intelligence, automated text classification, machine learning, systematic review

## Abstract

**Background:**

A growing body of literature has detailed the use of qualitative analyses to measure the therapeutic processes and intrinsic effectiveness of psychotherapies, which yield small databases. Nonetheless, these approaches have several limitations and machine learning algorithms are needed.

**Objective:**

The objective of this study is to conduct a systematic review of the use of machine learning for automated text classification for small data sets in the fields of psychiatry, psychology, and social sciences. This review will identify available algorithms and assess if automated classification of textual entities is comparable to the classification done by human evaluators.

**Methods:**

A systematic search was performed in the electronic databases of Medline, Web of Science, PsycNet (PsycINFO), and Google Scholar from their inception dates to 2021. The fields of psychiatry, psychology, and social sciences were selected as they include a vast array of textual entities in the domain of mental health that can be reviewed. Additional records identified through cross-referencing were used to find other studies.

**Results:**

This literature search identified 5442 articles that were eligible for our study after the removal of duplicates. Following abstract screening, 114 full articles were assessed in their entirety, of which 107 were excluded. The remaining 7 studies were analyzed. Classification algorithms such as naive Bayes, decision tree, and support vector machine classifiers were identified. Support vector machine is the most used algorithm and best performing as per the identified articles. Prediction classification scores for the identified algorithms ranged from 53%-91% for the classification of textual entities in 4-7 categories. In addition, 3 of the 7 studies reported an interjudge agreement statistic; these were consistent with agreement statistics for text classification done by human evaluators.

**Conclusions:**

A systematic review of available machine learning algorithms for automated text classification for small data sets in several fields (psychiatry, psychology, and social sciences) was conducted. We compared automated classification with classification done by human evaluators. Our results show that it is possible to automatically classify textual entities of a transcript based solely on small databases. Future studies are nevertheless needed to assess whether such algorithms can be implemented in the context of psychotherapies.

## Introduction

The intrinsic effectiveness of psychotherapies is generally measured through semistructured interviews or self-reported questionnaires [1-3]. However, these instruments have limitations in relation to constructs that can be set a priori, for which there are standardized measures available. To assess the intrinsic effectiveness of psychotherapies (the psychotherapeutic process itself), an increasing number of research teams have started to use qualitative methods. Although these approaches have inherent biases (eg, data analysis subjectivity), mathematical algorithms can be used to reduce such biases. Furthermore, assessment of a psychotherapy’s intrinsic effectiveness usually refers to an assessment of a patient’s characteristics and the therapeutic process [4]. Studies often use therapy session transcripts to qualitatively evaluate psychotherapies [5]. For in-person therapies, transcriptions are often time-consuming and classifying therapeutic interactions under various themes (labels) for analysis is even more demanding. Machine learning is a potential solution to reduce the amount of labor-intensive work required [6]. With the increasing development of new psychotherapies for various psychopathologies, there is a higher need for tools to measure and understand their effectiveness.

Text mining is one of the few techniques used in psychiatry to derive data from the large number of interactions that occur during therapy sessions [7]. One such technique is the use of artificial intelligence by means of machine learning. It is currently being used in many areas in the medical field, ranging from surgical procedure analyses to medical diagnostics [8]. When attempting to classify textual entities from medical fields into various categories, the text is often classified into a few categories. This can be done by applying a set of rules to an algorithm to be used for classification and is usually facilitated by the nature of the entity being classified (eg, signs and symptoms relating to a particular diagnosis or treatment) [9]. Classification of therapeutic interactions can be tricky considering the vast array of information associated with the therapy itself, the ability of the patient to communicate, and the context in which the therapy is being conducted [10]. This leads to transcripts that may vary widely from patient to patient; therefore, the information is less directly interpretable than medical records or results. In relevant fields where such data is usually used for research, such as psychiatry and psychology, the use of machine learning in the context of text mining in psychotherapy has been limited [11]. Many algorithms are readily available to conduct automated text classification [12]. Simple probabilistic mathematical algorithms (ie, naive Bayesian probability algorithms) as well as more complex ones (ie, neural networks) are available via open access libraries on the web [13]. Machine learning algorithms often need large databases to adequately classify new data by creating training sets and testing sets [14-16]. Large databases, such as some seen in the field of internet-enabled cognitive behavioral therapy, are required for complex machine learning algorithms to adequately learn and classify new information [1]. However, in-person therapies often yield databases that are smaller than the ones generated by internet-enabled cognitive behavioral therapy because of the need for human-driven transcriptions. This creates a need to find potential algorithms that can operate on small databases [17,18]. A machine learning algorithm applicable for small databases is therefore needed for such cases.

The objective of this study is to conduct a systematic review of the use of machine learning for automated text classification for small databases in the fields of psychiatry, psychology, and social sciences to determine the best algorithm for automatically classifying the content of psychotherapy transcripts. This would provide an interesting solution for automated therapy annotations in the context of qualitative analysis and could generate data to enable the evaluation of therapeutic processes.

## Methods

### Search Strategies

A systematic search was performed in the electronic databases of Medline, Web Of Science, PsycNet (PsycINFO), and Google Scholar from their inception dates until 2021 using text words and indexing (MeSH) terms with keywords that were inclusive for the fields of psychiatry (eg, psychiatric, psychiatry), psychology (eg, psychology, psychotherapy, neuropsychology) and social sciences (eg, social science) and machine learning. Additional records identified through cross-referencing were used to find other studies. The fields of psychiatry, psychology, and social sciences were selected as they include a vast array of textual entities in the domain of mental health that can be reviewed. A complete electronic search strategy is available in Multimedia Appendix 1. The search methodology was developed by the corresponding author and a librarian specialized in mental health at the Institut universitaire en santé mentale de Montréal. Searches were completed by AH and cross-validated by MB in May 2021. No setting, date, or geographical restrictions were applied. Searches were limited to English- or French-language sources.

### Study Eligibility

Studies were included if they met the following criteria: (1) classification in various data categories of textual entities (eg, medical records, letters, transcripts); (2) the study was conducted in the fields of psychiatry, psychology, or social sciences; (3) automated classification of text was conducted in more than 2 data categories (text was classified in more than two features); (4) automated text classification was conducted by machine learning (either supervised or unsupervised algorithms); and (5) the number of elements in the database used was less than 10,000, which corresponds to a small database. Although there is no consensus on what a small database is, we defined a small database as one that had a maximum of 10,000 items since 5000-10,000 items have been referred to as small samples in prior studies [19-21]. Studies that use a combination of many algorithms, instead of a single algorithm, were also included. Unpublished literature was excluded as well as studies using artificial intelligence algorithms outside the scope of machine learning.

### Data Extraction

Data were extracted with a standardized form and cross-verified for consistency and integrity by two authors, AH and MB. Information such as size of the database, number of classification categories, algorithms used, prediction success rate (in %), and interjudge agreement were recorded.

## Results

### Description of Studies

**Figure 1:**
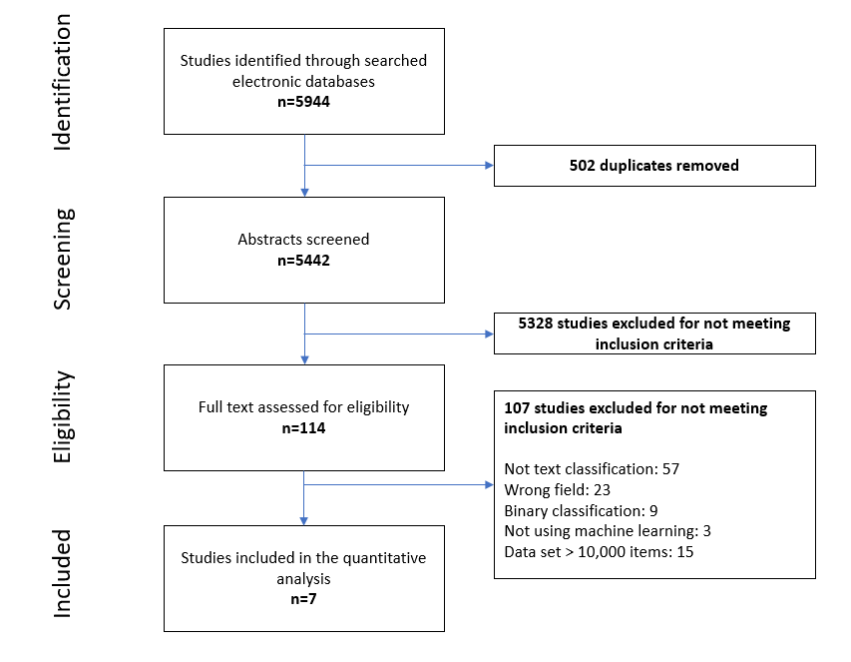
Flowchart depicting the process of study selection.

Our systematic review assessed studies that used machine learning to classify text in the fields of psychiatry, psychology, and social sciences. This literature search identified 5442 articles that were eligible for our study after the removal of duplicates. Following abstract screening, 114 full articles were assessed in their entirety, of which 107 were excluded. The remaining 7 studies were analyzed. The flowchart for the inclusion of studies in this systematic review is found in Figure 1. The details of the studies are provided in Multimedia Appendix 2. Notably, a limited number of articles on automated text classification with small databases were found. Studies that met inclusion criteria reported different types of documents used for automated annotation. Social medical content, such as forum posts in the study by Yu et al [22] and Twitter entries in the study by Balakrishnan et al [23] generated the largest data sets (5000 and 5453 items, respectively). Those textual entities consisted of complete or partial sentences manually written by users and were annotated in their entirety. The remaining types of documents were mainly medical records completed by physicians or health science professionals. No image or mathematical data were classified by the algorithms as part of these studies.

### Algorithms

#### Overview

Several algorithms have been used on the presented textual entities. Naive Bayes classifier, decision tree–based algorithms, support vector machine (SVM) classifiers, and combinations of multiple algorithms were the main strategies used by the included studies. The number of categories for text classification ranged from 4-7 and overall precision classification ranged from 77.0%-91.8%. For the studies that included multiple algorithms, SVM-based algorithms demonstrated the best accuracy in 5 of 7 studies.

#### Naive Bayes Classifier

A naive Bayes classifier is a probabilistic-based classifier that makes use of Bayes’ theorem to classify items into different categories [12]. This type of classifier achieves average performance in the context of supervised learning [24]. This type of algorithm is advantageous when little data is available as it can be optimally parameterized in the event of a small data set [25]. This algorithm assumes that there is independence between the predictors. For text classification, Balakrishnan et al [23] outlined that this algorithm works best when using each word as a variable that needs to be classified.

#### Decision Tree–Based Classifiers

Decision tree–based classifiers are nonparameterized; they are supervised learning methods that can be used to classify items [26]. Observations about an item are represented as branches and conclusions about an item's value (score) are represented as leaves [27]. Splitting across the different branches is based on defined rules according to the categories used to classify the items. In text classification, the general idea is that every piece of text being classified is split across the branches until it reaches a leaf (category) based on probabilistic rules set by the designer of the tree [27].

#### SVM Classifiers

SVM classifiers can be used in both supervised and unsupervised learning contexts. In simple terms, these classifiers use the concept of a hyperplane that divides a data set into classes. A hyperplane in an n-dimensional Euclidean space is a flat, n–1 dimensional subset of that space that divides the space into two disconnected parts [28]. The items in the data set are considered as data points on the hyperplane. The item being classified is therefore categorized in one of the disconnected parts.

### Outcomes

In the 7 identified studies, SVM classifiers and algorithms combined with SVM classifiers tended to achieve the best prediction score (in %) as compared to other algorithms for small data sets. Studies by Zolnoori et al [29], Singh et al [30], and Yu et al [22] reported prediction scores of SVM classifiers that were superior to other classifiers for their data sets. Their precision scores ranged from 77%-90%. Only 3 studies attempted to compare the classification done by the classifiers with human annotators. The statistics used to assess these automated annotations were κ and pairwise agreements. The interrater agreement of these studies was comparable to interrater agreements for annotation done by human annotators; the κ scores were 0.84 [23], 0.67 [30], and 0.86 [29], respectively.

## Discussion

### Review of Findings

In this study, we conducted a systematic review to identify potential algorithms that could be useful for small databases for the automatic annotation of unannotated interview transcripts from the field of psychotherapy. The systematic review we conducted demonstrated that limited literature exists on the subject. However, few algorithms displayed sufficient accuracy when performing text classification on small databases. SVM classifiers tended to display the best accuracy in the context of small databases.

Compared to other reviews on the subject, this study highlights algorithms being used in the context of small data sets, which is consistent with the reality of studies of therapies [31], as transcribing therapy sessions is time-consuming and demanding. Regarding novel therapy developments, such as virtual reality–based therapy, this is even more needed considering the small number of patients that have received these treatments so far [32]. Therapy usually involves a wider range of words and contextual sentences compared to other areas of medicine where specific words (eg, symptoms, signs) can be used to facilitate classification. Therefore, it is not surprising to see that this systematic review identified algorithms that differ from those that are widely used in other medical fields. For example, Srivastava et al [33] reviewed the efficiency of different text classifiers in the context of social media posts referring to medical content. They found that a multilayer perceptron–based neural network performed best in their study as compared to a SVM classifier. Another study, conducted by Visveswaran and colleagues [34], identified convolutional long short-term memory neural networks as the best at predicting vaping habits. This can be explained by the fact that most classifiers are combined with a vectorizer when used to classify textual entities. A vectorizer transforms text into a meaningful number vector that can then be used by classifiers [35]. Considering that classification of textual entities to identify a specific diagnosis or medical condition usually requires specific terms that pertain to the diagnosis or condition, vectors tend to discriminate better between the textual entities of these fields [36]. This is usually not the case with therapy transcripts in the context of analysis of the psychotherapeutic process as this analysis often requires a larger array of categories that can sometime overlap.

In contrast with other types of medical data—such as imagery or numerical entities (eg, laboratory results)—where neural networks seem to be the most used class of algorithms for classification, textual classification appears to be performed with a more restricted number of classifiers [37]. This can be explained by the fact that text classification requires additional considerations. Automated classifications lack the ability to interpret a sentence out of a given context (eg, a therapeutic session), while the meaning of a sentence could change based on the context. Another complexity is that words can refer to different entities based on the sociocultural context. Therefore, considering such complexities can require further parameterizations and considerations, which may also explain why, in the identified studies, the same algorithm used on data sets of a similar size could have a diverging predictive score.

Consistent with our findings, linear SVM classifiers tend to be regarded as one of the best text classifying algorithms in the literature [38]. Many types of classifiers are available, but it appears that only a few are consistently used for the classification of textual entities [26]. This is consistent with our review, as the identified studies tended to use similar strategies when classifying textual entities. A recent literature review on data classification of clinical text data explains this phenomenon by the fact that there is a bottleneck of annotations in the context of supervised learning [39].

### Limitations

This systematic review of literature focuses on the fields of psychiatry, psychology, and social sciences to reflect the type of textual entities usually found in therapy transcripts. A limitation of this study is the small number of classification algorithm studies published in these fields. As this is an emerging domain, the number of studies on the topic should increase in the future.

### Conclusions

Machine learning can be beneficial for the field of psychiatry. Automated text classification for psychotherapy is a promising avenue to generate quantitative and qualitative data in an efficient way to make the data readily available for analyses. SVM classifiers appear to be preferred over other types of classifiers in the context of small databases. Using such classifiers could be useful in the evaluation of therapeutic processes of novel therapies where data are limited. Nevertheless, the limited number of articles found on the subject outlines the need for more development in this field, especially regarding the use of such classifiers in the domain of mental health.

## Appendix

Multimedia Appendix 1 Electronic search strategy for the systematic review conducted.

Multimedia Appendix 2 Detailed results of the systematic review study selection.

## Abbreviations

SVM: support vector machine

## References

[1] Ewbank MP, Cummins R, Tablan V, Bateup S, Catarino A, Martin AJ, Blackwell AD (2020). Quantifying the Association Between Psychotherapy Content and Clinical Outcomes Using Deep Learning. JAMA Psychiatry.

[2] Cook SC, Schwartz AC, Kaslow NJ (2017). Evidence-Based Psychotherapy: Advantages and Challenges. Neurotherapeutics.

[3] Hill C, Chui H, Baumann E, Kazdin AE (2016). Revisiting and reenvisioning the outcome problem in psychotherapy: An argument to include individualized and qualitative measurement. Methodological issues and strategies in clinical research (4th ed).

[4] Szymańska A, Dobrenko K, Grzesiuk L (2017). Characteristics and experience of the patient in psychotherapyand the psychotherapy’s effectiveness. A structural approach. Psychiatr Pol.

[5] Perepletchikova F (2011). On the topic of treatment integrity. Clinical Psychology: Science and Practice.

[6] Sebastiani F (2002). Machine learning in automated text categorization. ACM Comput Surv.

[7] Abbe A, Grouin C, Zweigenbaum P, Falissard B (2016). Text mining applications in psychiatry: a systematic literature review. Int J Methods Psychiatr Res.

[8] Khalid S, Goldenberg M, Grantcharov T, Taati B, Rudzicz F (2020). Evaluation of Deep Learning Models for Identifying Surgical Actions and Measuring Performance. JAMA Netw Open.

[9] Tang S, Chappell GT, Mazzoli A, Tewari M, Choi SW, Wiens J (2020). Predicting Acute Graft-Versus-Host Disease Using Machine Learning and Longitudinal Vital Sign Data From Electronic Health Records. JCO Clinical Cancer Informatics.

[10] Høglend P (2014). Exploration of the patient-therapist relationship in psychotherapy. Am J Psychiatry.

[11] Durstewitz D, Koppe G, Meyer-Lindenberg A (2019). Deep neural networks in psychiatry. Mol Psychiatry.

[12] Gupta A, Katarya R (2020). Social media based surveillance systems for healthcare using machine learning: A systematic review. Journal of Biomedical Informatics.

[13] Vora S, Yang H (2017). A Comprehensive Study of Eleven Feature Selection Algorithms and their Impact on Text Classification. 2017 Computing Conference.

[14] Deo RC (2015). Machine Learning in Medicine. Circulation.

[15] Cao H, Meyer-Lindenberg A, Schwarz E (2018). Comparative Evaluation of Machine Learning Strategies for Analyzing Big Data in Psychiatry. Int J Mol Sci.

[16] Kowsari K, Jafari Meimandi K, Heidarysafa M, Mendu S, Barnes L, Brown D (2019). Text Classification Algorithms: A Survey. Information.

[17] Hämäläinen W, Vinni M (2006). Comparison of Machine Learning Methods for Intelligent Tutoring Systems. Intelligent Tutoring Systems.

[18] Wanigasekara C, Swain A, Nguang SK, Prusty BG (2018). Improved Learning from Small Data Sets Through Effective Combination of Machine Learning Tools with VSG Techniques. International Joint Conference on Neural Networks.

[19] Shiner B, D'Avolio L, Nguyen T, Zayed M, Watts B, Fiore L (2012). Automated classification of psychotherapy note text: implications for quality assessment in PTSD care. J Eval Clin Pract.

[20] Slonim N, Tishby N (2001). The Power of Word Clusters for Text Classification. 23rd European Colloquium on Information Retrieval Research.

[21] Joachims T (1999). Transductive inference for text classification using support vector machines. ICML.

[22] Yu L, Chan C, Lin C, Lin I (2011). Mining association language patterns using a distributional semantic model for negative life event classification. J Biomed Inform.

[23] Balakrishnan V, Khan S, Arabnia HR (2020). Improving cyberbullying detection using Twitter users’ psychological features and machine learning. Computers & Security.

[24] Zhang W, Gao F (2011). An Improvement to Naive Bayes for Text Classification. Procedia Engineering.

[25] Huang Y, Li L (2011). Naive Bayes classification algorithm based on small sample set.

[26] Vijayan VK, Bindu KR, Parameswaran L (2017). A comprehensive study of text classification algorithms.

[27] Kamiński B, Jakubczyk M, Szufel P (2018). A framework for sensitivity analysis of decision trees. Cent Eur J Oper Res.

[28] Noble WS (2006). What is a support vector machine?. Nat Biotechnol.

[29] Zolnoori M, Fung KW, Patrick TB, Fontelo P, Kharrazi H, Faiola A, Wu YSS, Eldredge CE, Luo J, Conway M, Zhu J, Park SK, Xu K, Moayyed H, Goudarzvand S (2019). A systematic approach for developing a corpus of patient reported adverse drug events: A case study for SSRI and SNRI medications. J Biomed Inform.

[30] Singh V, Shrivastava U, Bouayad L, Padmanabhan B, Ialynytchev A, Schultz S (2018). Machine learning for psychiatric patient triaging: an investigation of cascading classifiers. J Am Med Inform Assoc.

[31] Hartmann J, Huppertz J, Schamp C, Heitmann M (2019). Comparing automated text classification methods. International Journal of Research in Marketing.

[32] Fodor LA, Coteț CD, Cuijpers P, Szamoskozi Ș, David D, Cristea IA (2018). The effectiveness of virtual reality based interventions for symptoms of anxiety and depression: A meta-analysis. Sci Rep.

[33] Srivastava SK, Singh SK, Suri JS (2018). Healthcare Text Classification System and its Performance Evaluation: A Source of Better Intelligence by Characterizing Healthcare Text. J Med Syst.

[34] Visweswaran S, Colditz JB, O'Halloran P, Han N, Taneja SB, Welling J, Chu K, Sidani JE, Primack BA (2020). Machine Learning Classifiers for Twitter Surveillance of Vaping: Comparative Machine Learning Study. J Med Internet Res.

[35] Shahmirzadi O, Lugowski A, Younge K (2017). Text Similarity in Vector Space Models: A Comparative Study.

[36] Khattak FK, Jeblee S, Pou-Prom C, Abdalla M, Meaney C, Rudzicz F (2019). A survey of word embeddings for clinical text. J Biomed Inform.

[37] Yadav SS, Jadhav SM (2019). Deep convolutional neural network based medical image classification for disease diagnosis. J Big Data.

[38] Agnihotri D, Verma K, Tripathi P (2017). An automatic classification of text documents based on correlative association of words. J Intell Inf Syst.

[39] Spasic I, Nenadic G (2020). Clinical Text Data in Machine Learning: Systematic Review. JMIR Med Inform.

