# Supplementary material for: Use of Automated Thematic Annotations for Small Data Sets in a Psychotherapeutic Context: Systematic Review of Machine Learning Algorithms
Source: JMIR Ment Health. 2021 Oct 22;8(10):e22651. doi: 10.2196/22651 (PMC8571689; doi:10.2196/22651)
Supplement: Multimedia Appendix 1 [file mental_v8i10e22651_app1.docx]

**Supplementary Online Content**

Hudon, A., Beaudoin, M, Phraxayavong, K., Dellazizzo, L., Potvin, S., Dumais, A. Automated thematic classification: Systematic review of existing machine learning algorithms for small databases to assess psychotherapeutic process

**Multimedia Appendix 1.** Electronic search strategy for the systematic review conducted.

**Multimedia Appendix 1. Electronic search strategy for the systematic review conducted.**

| **Database; Search** | **Search Terms** |
| --- | --- |
|  |  |
| PubMed; k= 2868 | ("Machine Learning"[Mesh] OR "Natural Language Processing"[Mesh] OR "Data Mining"[Mesh] OR "Machine learning"[TIAB] OR "deep learning"[TIAB] OR "text mining"[TIAB] OR "data mining"[TIAB] OR "learning algorithm"[TIAB] OR "learning algorithms"[TIAB] OR "classification algorithm"[TIAB] OR "classification algorithms"[TIAB] OR "language processing"[TIAB] OR "text analysis"[TIAB]) AND Psychiatric[TIAB] OR Psychiatry[TIAB] OR Psychotherapy[TIAB] OR psychotherapies[TIAB] OR therapy[TIAB] OR therapies[TIAB] OR Psychology[TIAB] OR Neuropsychology[TIAB] OR Psychological[TIAB] OR neuropsychological[TIAB] OR "Social science"[TIAB] OR "social sciences"[TIAB]) |
| Web of Science; k= 2736 | (Psychiatric OR Psychiatry OR Psychotherapy OR Psychotherapies OR Therapy OR Therapies OR Psychology OR Neuropsychology OR Psychological OR Neuropsychological OR Social science OR Social sciences) AND TS= (Machine learning OR Deep learning OR text mining OR data mining OR learning algorithm OR learning algorithms OR classification algorithm OR classification algorithms OR language processing OR text analysis) |
| PsychInfo; k = 4 | exp machine learning/ OR exp data mining/ OR exp automated information processing/ OR ("Machine learning" or "deep learning" or "text mining" or "data mining" or "learning algorithm" or "learning algorithms" or "classification algorithm" or "classification algorithms" or "language processing" or "text analysis").ab. or ("Machine learning" or "deep learning" or "text mining" or "data mining" or "learning algorithm" or "learning algorithms" or "classification algorithm" or "classification algorithms" or "language processing" or "text analysis").ti. AND (Psychiatric or Psychiatry or Psychotherapy or psychotherapies or Therapy or therapies or Psychology or Neuropsychology or Psychological or neuropsychological or "Social science" or "social sciences").ab. or (Psychiatric or Psychiatry or Psychotherapy or psychotherapies or Therapy or therapies or Psychology or Neuropsychology or Psychological or neuropsychological or "Social science" or "social sciences").ti. |
| Google Scholar; k= 336 | (allintitle:"Machine learning" OR "Text mining" OR "classification algorithm" OR "language processing" OR "text analysis") AND ("Psychiatry" OR "Psychiatric" OR "Psychology" OR "Social sciences" OR "social sciences "OR "Neuropsychology" OR "therapy" OR "therapies" OR "psychotherapy" OR "psychotherapies") AND ("text classification" OR "text processing") |
