# Supplementary material for: Use of Automated Thematic Annotations for Small Data Sets in a Psychotherapeutic Context: Systematic Review of Machine Learning Algorithms
Source: JMIR Ment Health. 2021 Oct 22;8(10):e22651. doi: 10.2196/22651 (PMC8571689; doi:10.2196/22651)
Supplement: Multimedia Appendix 2 [file mental_v8i10e22651_app2.docx]

**Supplementary Online Content**

Hudon, A., Beaudoin, M, Phraxayavong, K., Dellazizzo, L., Potvin, S., Dumais, A. Automated thematic classification: Systematic review of existing machine learning algorithms for small databases to assess psychotherapeutic process

**Multimedia Appendix 2.** Systematic review study selection detailed results.

This supplementary material has been provided by the authors to give readers additional information about their work.

| **Studies** | **Dataset size** | **Datatype** | **# categories** | **Algorithms used** | **Algorithm with highest accuracy** | **Precision** | **Recall** | **Prediction Statistic** | **Prediction Score (%)** | **Inter-judge agreement statistic** | **Inter-judge agreement statistic** |
| --- | --- | --- | --- | --- | --- | --- | --- | --- | --- | --- | --- |
| [22] Balakrishnan V, Khan S, Arabnia H.R., 2020 | 5453 | Twitter entries | 4 | NB, RF, J48 | J48 (DT based)^a^ | N/A | 0.97 | F1-Score | 91.88 | Kappa | 0.84 |
| [23] Zolnoori et al., 2019 | 891 | drug review posts | 6 | SVM , RF, NB, LR | SVM | 0.91 | 0.912 | F1-Score | 90.06 | Pairwise agreement | 0.86 |
| [24] Karystianis et al, 2018 | 541 | psychiatric records | 4 | Rule-based method, NN | Rule-based method | N/A | N/A | MAE | 80.1 | N/A | N/A |
| [25] Singh et al., 2018 | 325 | Initial psychiatric assessment record | 7 | LSVM and other algorithms | One-class-at-a-time (LSVM and others) ^b^ | N/A | N/A | INMAE | 77 | Kappa | 0.67 |
| [26] Clark et al., 2017 | 600 | Patient clinical notes | 4 | MLP and MLR classifiers | MLP with a 20% drop-rate N/A | N/A | N/A | MAE | 77.86 | N/A | N/A |
| [27] Dai H.J., Jonnagaddala J, 2018 | 649 | Patient records | 4 | CNN, SVM, C4,5, NB | CNN overall SVM for 2 categories | N/A | N/A | MAE | Mean: 53.9 Normalized :78.5 | N/A | N/A |
| [28] Yu et al., 2011 | 5000 | Forum posts | 5 | NB, C4,5 , TAN, SVM | SVM | N/A | N/A | F1-Score | 79.5 to 81.9 | N/A | N/A |

**Multimedia Appendix 2. Systematic review study selection detailed results.**

*Abbreviations: NB = Naïve Bayes, LR = Logistic Regression RF= Random forest, DT= Decision Tree, SVM = Support vector machine, NN = Neural Network, LSVM = Linear support vector machine, MLP = Multiple layered platform, CNN= Convolutional Neural Network, TAN= Tree Augmented Naïve Bayes. ^a^J48 is a decision-tree based algorithm often combined with an SVM. ^b^ In the One-class-at-a-time cascading algorithm, linear SVM was the best performing algorithm with an accuracy of 61%.*
